# Prediction of flexible/rigid regions from protein sequences using k-spaced amino acid pairs

**DOI:** 10.1186/1472-6807-7-25

**Published:** 2007-04-16

**Authors:** Ke Chen, Lukasz A Kurgan, Jishou Ruan

**Affiliations:** 1 Department of Electrical and Computer Engineering, University of Alberta, Edmonton, AB, Canada; 2 Chern Institute of Mathematics, College of Mathematical Science and LPMC, Nankai University, Tianjin 300071, PCR

## Abstract

**Background:**

Traditionally, it is believed that the native structure of a protein corresponds to a global minimum of its free energy. However, with the growing number of known tertiary (3D) protein structures, researchers have discovered that some proteins can alter their structures in response to a change in their surroundings or with the help of other proteins or ligands. Such structural shifts play a crucial role with respect to the protein function. To this end, we propose a machine learning method for the prediction of the flexible/rigid regions of proteins (referred to as FlexRP); the method is based on a novel sequence representation and feature selection. Knowledge of the flexible/rigid regions may provide insights into the protein folding process and the 3D structure prediction.

**Results:**

The flexible/rigid regions were defined based on a dataset, which includes protein sequences that have multiple experimental structures, and which was previously used to study the structural conservation of proteins. Sequences drawn from this dataset were represented based on feature sets that were proposed in prior research, such as PSI-BLAST profiles, composition vector and binary sequence encoding, and a newly proposed representation based on frequencies of k-spaced amino acid pairs. These representations were processed by feature selection to reduce the dimensionality. Several machine learning methods for the prediction of flexible/rigid regions and two recently proposed methods for the prediction of conformational changes and unstructured regions were compared with the proposed method. The FlexRP method, which applies Logistic Regression and collocation-based representation with 95 features, obtained 79.5% accuracy. The two runner-up methods, which apply the same sequence representation and Support Vector Machines (SVM) and Naïve Bayes classifiers, obtained 79.2% and 78.4% accuracy, respectively. The remaining considered methods are characterized by accuracies below 70%. Finally, the Naïve Bayes method is shown to provide the highest sensitivity for the prediction of flexible regions, while FlexRP and SVM give the highest sensitivity for rigid regions.

**Conclusion:**

A new sequence representation that uses k-spaced amino acid pairs is shown to be the most efficient in the prediction of the flexible/rigid regions of protein sequences. The proposed FlexRP method provides the highest prediction accuracy of about 80%. The experimental tests show that the FlexRP and SVM methods achieved high overall accuracy and the highest sensitivity for rigid regions, while the best quality of the predictions for flexible regions is achieved by the Naïve Bayes method.

## Background

The flexibility of protein structures is often related to protein function. Some proteins alter their tertiary (3D) structures due to a change of surroundings or as a result of interaction with other proteins [1-3]. For instance, the GTP-binding proteins adopt an active conformation when binding with GTP, and shift to inactive conformation when GTP is hydrolyzed to GDP [4,5]. Motor proteins shift their structure among multiple conformations [6,7], while many carrier proteins embedded in a membrane transport small molecules by executing structural changes [8,9]. In short, the structural flexibility that allows shifting between two or more structures is a crucial characteristic for numerous proteins that are involved in many pathways [10,11]. Although proteins can shift structure among several conformations, some of their segments, referred to as conserved domains, preserve the structure in all of the conformations [12,13]. In fact, many proteins that can change their conformations can be divided into the rigid (conserved) region(s) and the flexible region(s), in some cases referred to as linkers, which serve to link and adjust the relative location of the conserved domains. Upon the arrival of an external signal, such as a change in surroundings or a binding of another molecule/protein, the flexible region allows the protein to respond by changing its conformation. In other words, the flexible linker is essential for a protein to maintain flexibility and the corresponding function [14,15].

Additionally, the flexible linker and the rigid domain should be factored in when performing 3D protein structure prediction. Protein is a complex system that can be described by an accurate energy-based model [16,17]. However, due to the large numbers of atoms involved in the protein folding, and the resulting large amount of calculations, protein structures cannot be directly calculated (predicted) based on the existing mechanical models employed by current supercomputers. A natural solution to this problem is to apply a divide-and-conquer approach, in which a large protein is divided into several structurally conserved domains, and each of the domains is predicted separately [18,19]. A number of methods can be used for the prediction of protein domains [20]. At the same time, the remaining (except the conserved domains) protein regions that are located between domain borders may be flexible, and knowledge of their flexibility would be beneficial to accurately predict the overall tertiary structure.

The knowledge of the flexible/rigid regions would also allow us to gain insights into the process of protein folding. Biological experiments and theoretical calculation have shown that the natural conformation of proteins is usually associated with the minimum of the free energy [21-23]. However, the overall process that leads to the final, stable conformation is still largely unknown. Udgaonkar and Baldwin propose a framework model for protein folding [24]. Their theory states that peptides of about 15 amino acids (AAs) firstly fold into helices and strands, and then these secondary structures are assembled together to form the molecule. The hydrophobic collapse model proposed by Gutin and colleagues assumes the initial condensation of hydrophobic elements that gives rise to compact states without secondary structures. The development of native-like tertiary interactions in the compact states prompts the subsequent formation of the stable secondary structures [25]. A recent paper by Sadqui and colleagues shows the detailed process of the unfolding of the downhill protein BBL from Escherichia coli, atom by atom, starting from a defined 3D structure [26]. However, the detailed process of folding of most proteins is still under investigation. The proteins with flexible 3D-structures may provide some hints since the conserved regions should fold separately from the flexible regions to eventually get linked into a stable (and potentially susceptible to structural change) structure.

**Figure 1:**
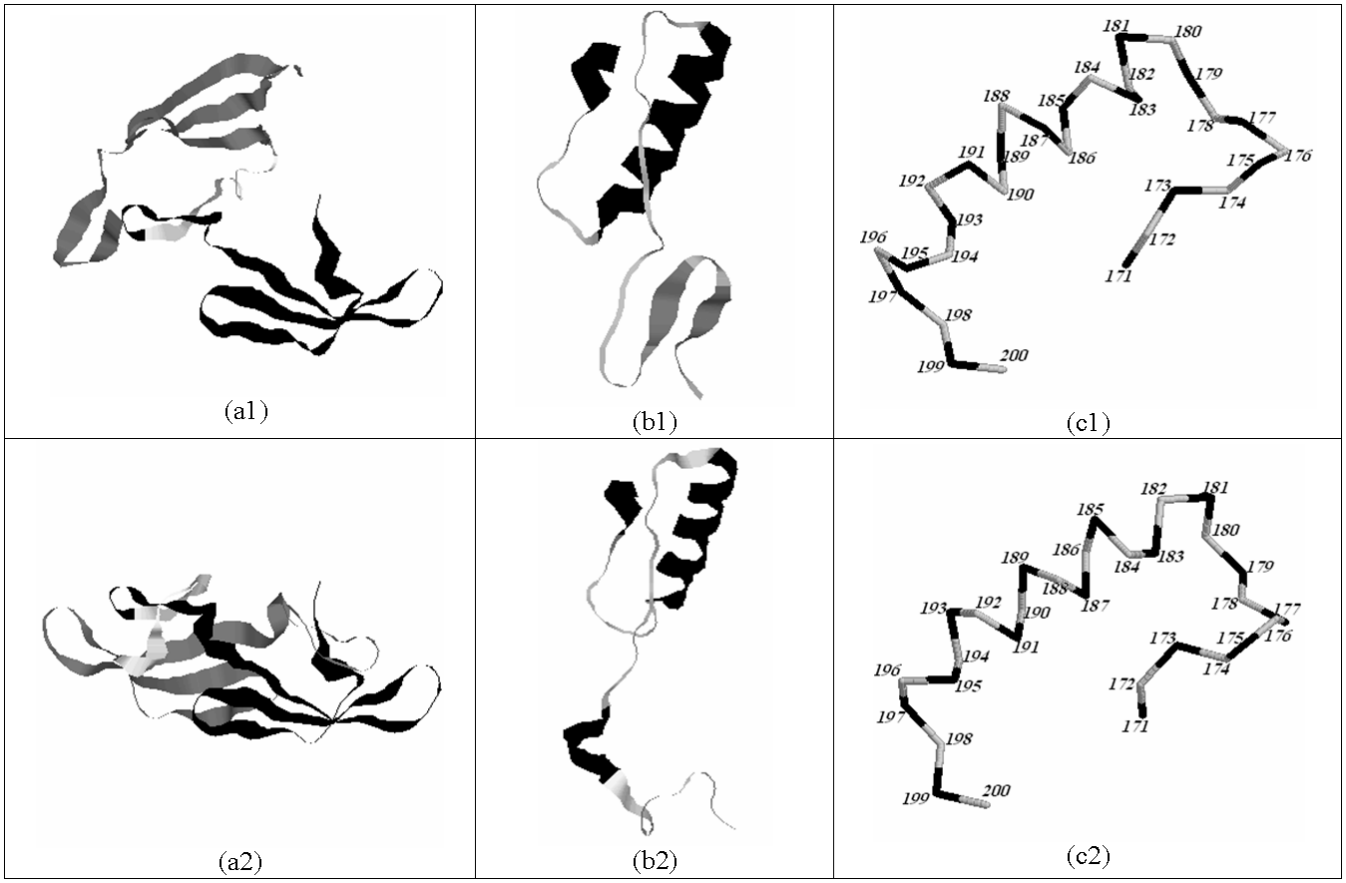
**Examples of the three types of flexible regions**. 1) Pair (a1) and (a2) is an example of *rotating *regions. (a1) is chain A of protein 1l5e from Leu1 to Tyr100 and (a2) is protein 2ezm from Leu1 to Tyr 100. Both fragments share the same sequence, are build from two domains (colored gray and black) that also share the same structure, but the structures of the linkers (colored in light gray) are different. 2) Pair (b1) and (b2) is an example of regions with *missing *secondary structure. (b1) is chain A of protein 1ikx from Glu224 to Leu279 and (a2) is chain B of protein 1ikx f from Glu1224 to Leu1279. Both fragments share the same sequence. The Phe227 to Leu234 in (b1) forms a strand, while it forms a coil in (b2). 3) Pair (c1) and (c2) is an example of *disarranging *regions. (c1) is chain A of protein 1ffx from Ile171 to Cys200 and (c2) is chain A of protein 1jff from Ile171 to Cys200. The fragments share the same sequence, and have similar overall 3D-structure and secondary structure. At the same time, the URMSD between these two structures is larger than 0.8 since the middle region between 180Ala and 192His is disarranged. The spatial packing of the corresponding AAs is different for this region.

Gerstein's group has done a significant amount of work on the related subject of classification of protein motions [27,28]. They proposed two basic mechanisms of protein motion, *hinge *and *shear*, which depend on whether or not a continuously maintained interface (between different, rigid parts of protein) is preserved through the protein's motion. The shear mechanism is a kind of a small, sliding motion in which a protein preserves a well-packed interface. In contrast, hinge motion is not constrained by maintaining the interface, and this motion usually occurs in proteins with domains connected by linkers. They also defined other possible motions, and among them those that involve a partial refolding of a protein and thus result in significant changes in the overall protein structure. This paper does not study protein motion. Instead, we aim at finding protein-sequence regions that are flexible and hence which constitute the interface between the rigid regions. In our recent work, we performed a comprehensive, quantitative analysis of the conservation of protein structures stored in PDB, and we found three distinct types of the flexible regions, namely *rotating*, *missing*, and *disarranging *[12]. The *rotating *region of a protein sequence is related to the hinge motion, i.e., it usually contains a linker which is located between two domains. On the other hand, the *missing *and *disarranging *regions correspond to the types of motions that involve a partial refolding. The *missing *region is associated with changes in the local, secondary structure conformations, which may also lead to different tertiary structures. For instance, given two structures that share the same sequence, some regions of one structure may form a helix or a strand, while the same regions in the other structure may form an irregular coil. For the *disarranging *region, the overall 3D conformations of two identical underlying sequences are similar, but the packing of the residues is spatially shifted (disarranged) in some fragments of the region. We illustrate each of these three types of regions in Figure 1.

Since the regions that are *missing *a secondary structure are characterized by relatively small changes in the overall tertiary structure [12], in this paper we associate the flexibility of the protein structures with the two other types of flexible regions. Our aim is to perform prediction of the flexible regions using machine-learning methods that take as an input a feature based representation generated from the primary sequence. Several other research groups addressed similar prediction tasks, including prediction of regions undergoing conformational changes [29], prediction of intrinsically unstructured regions [30] and prediction of functionally flexible regions [31]. However, these contributions have different underlying goals, use different definitions of "flexible" regions (i.e., unstructured, undergoing conformation change, and functionally flexible), and apply different prediction models. Our goal is to classify each residue as belonging to either a flexible or a rigid region. The quality of the prediction is evaluated on a carefully designed (based on the rotations and disarrangements) set of 66 proteins using the accuracy, sensitivity, specificity and Matthews Correlation Coefficient (MCC) measures and an out-of-sample cross-validation test procedure. The methods section provides further details on the definition of the flexible regions and situates it with respect to the related research.

## Results and discussion

### Feature-based sequence representation

Four groups of features were compared, and the best set was selected to perform the prediction. The *composition vector*, *binary encoding *and *PSI-BLAST profile *representations are widely used in protein structure prediction including the structural class prediction, the secondary structure prediction and the cis/trans isomerization prediction. However, since these features were not designed for the prediction of flexible/rigid regions, a new representation, which is based on frequencies of *k-spaced residue pairs*, was proposed and compared with the other three representations. Due to a relatively large number of features, the binary encoding and the proposed representation were processed by two feature selection methods, which compute *linear correlation *and information gain based on *entropy *between each of the features and the predicted variable, i.e., rigidity/flexibility of the residues. The selection was performed using 10-fold cross validation to avoid overfitting. Only the features that were selected by a given method in all 10 folds were kept. While in general each of the two methods selects a different set of features, among the best 95 features selected by the entropy based method, 51 were also selected by the linear correlation based method.

Using the 10-fold cross validation, the proposed FlexRP method, which applies Logistic Regression and the proposed collocation based representation, which is processed using entropy based feature selection, was compared with four other prediction methods, i.e., Support Vector Machines (SVM), C4.5, IB1 and Naïve Bayes, which apply each of the four representations and two selection methods, see Table 1. The selected methods cover the major categories of machine learning algorithms, i.e., kernel methods, probabilistic methods, instance based learning and decision trees.

The proposed FlexRP method obtained the best, 79.5% accuracy, when compared with the other four methods, four representation and application of the two feature selection methods. The results for the two worst performing prediction methods, i.e., C4.5 and IB1, show relatively little differences in accuracy when the two feature selection methods are compared. On the other hand, results for the three best performing methods (FlexRP, SVM, and Naïve Bayes) show that using the entropy based feature selection results in the best accuracy of prediction when the proposed (best performing) representation is used. The results achieved by the proposed method are 1% and 3.5% better than two runner-up results achieved for the same representation and the SVM and Naïve Bayes classifiers, respectively. The results that apply other combinations of feature representations and selection methods are on average, over the three best methods, at least 4% less accurate. Therefore, entropy based selection not only reduces the dimensionality of the proposed representation, making it easier to implement and execute the method, but also results in improved accuracy. The superiority of the entropy based selection over the linear correlation based method can be explained by the type of features that constitute the proposed representation. The features take on discrete, integer values, and thus linear correlation coefficients, which prefer continuous values, are characterized by poorer performance.

Among the four sequence representations, the lowest average (over the five prediction methods) accuracy is achieved with the composition vector, while both PSI-BLAST profile and binary encoding give similar, second-best accuracies. The most accurate predictions are obtained with the proposed representation. Since the PSI-BLAST profile is one of the most commonly used representations, we also combined it with the features of the k-spaced AA pairs to verify whether this combination could bring further improvements. The corresponding experiments with the best performing three classifiers, i.e., FlexRP, Naïve Bayes and SVM, show that using both representations in tandem lowers the accuracy. The 10-fold cross validation accuracy equals 77.13%, 76.33%, and 72.99% for FlexRP, SVM, and Naïve Bayes, respectively. Finally, similar experiments that combine all four representations show a further drop in accuracy. The proposed, k-spaced residues based representation not only gives the best accuracy but it also uses the least number of features when compared to representations that combine multiple feature sets, and therefore this representation was used to perform the predictions.

A set of features used by the FlexRP method, which were selected using the best performing, entropy based selection method from the proposed representation, is given in Table 2. A Total of 95 features were selected. They correspond to threshold *IG*(*X*|*Y*) > 0.03, which gives the highest prediction accuracy for FlexRP and SVM. When varying the threshold to 0.035, 0.030 and 0.025, the corresponding accuracies for FlexRP are 78.79%, 79.51% and 78.96%, and for the SVM are 77.48%, 78.46% and 77.82%. Although this set of features may seem disordered, some interesting patterns can be found. For instance, the "LL" was selected as the 0-, 1-, 2- and 4-spaced AA pair, since Leucine has a strong tendency to form helices [32], and thus this pair may be characteristic for the rigid regions. The k-spaced "VI" pair is characteristic to formation of strands [32], and thus it may also be associated with the rigid regions. The k-spaced "GG" pair could indicate flexible regions since Glycine has a very small side chain (and thus it may be more flexible) and is shown to be mainly associated with coils [32]. At the same time, to the best of our knowledge, some of the other pairs cannot be currently explained. In general, the flexibility/rigidity of individual k-spaced pairs is associated with the arrangement of the corresponding side chains in 3D structure and their quality is supported by the relatively high accuracy of the methods that use this representation. We also performed a test, in which we accept all features that are selected in at least 9 out of the 10 cross-validation folds to investigate if inclusions of additional features can improve the results. The corresponding feature sets gave slightly lower accuracies. For the proposed representation, the corresponding accuracies dropped to 77.36% for SVM and to 78.99% for FlexRP.

### Optimization of the prediction of the flexible/rigid regions

Table 1 shows that among the five prediction methods, FlexRP, SVM, and Naïve Bayes are characterized by higher, on average by 5–8%, accuracies when compared with the remaining two machine learning methods. The three best methods achieve 76%–79% accuracy for the proposed representation that includes 95 features, while accuracy of the C4.5 and Naïve Bayes methods is below 69% and 67%, respectively. Therefore, the two worst methods were dropped, while the three best performing classifiers were optimized by exploration of their parameter space. As a result of the optimization, the FlexRP with a standard value of 10^-8 ^of ridge parameter for the Logistic Regression classifier, Naïve Bayes with kernel estimator for numeric attributes [33] and SVM with radial basis function based kernel and a corresponding gamma value of 0.22 (polynomial kernels of varying degrees were also considered) [34] were found as optimal. We note that the annotation of flexible/rigid regions is based on the current structures stored in PDB, and with the posting of new structures, the rigid regions could be reclassified as flexible (in contrast, the flexible regions could not be reclassified as rigid when assuming that the current data is correct). Therefore, maximization of prediction quality for flexible regions as a trade-off for reduced quality for rigid regions may be beneficial, given that the overall prediction accuracy does not decrease. This trade-off was implemented using a cost matrix with 1.0 misclassification cost for flexible regions and 0.6 cost for the rigid regions for the Naïve Bayes method. At the same time, the cost matrix was not found useful in the case of the other two methods.

The accuracies of the optimized prediction methods equal 79.51%, 78.41% and 79.22% for the FlexRP, Naïve Bayes and SVM, respectively. To provide a more comprehensive comparison of the achieved performance, additional measures such as sensitivity, specificity, the Matthews Correlation Coefficient (MCC) and the confusion matrix values (TP, FP, FN, and TN) are reported in Table 3, which lists 10-fold cross validation results for the three methods.

The optimization provides relatively marginal improvements. FlexRP method gives the best overall accuracy and high sensitivity and specificity for the rigid regions. SVM provides the best sensitivity for the rigid regions and the best specificity for the flexible regions, while Naïve Bayes gives the highest MCC and the highest sensitivity for the flexible regions. In summary, the proposed FlexRP method is shown to provide the most accurate prediction of flexible/rigid regions; however, Naïve Bayes based method provides more accurate prediction for the flexible regions.

**Figure 2:**
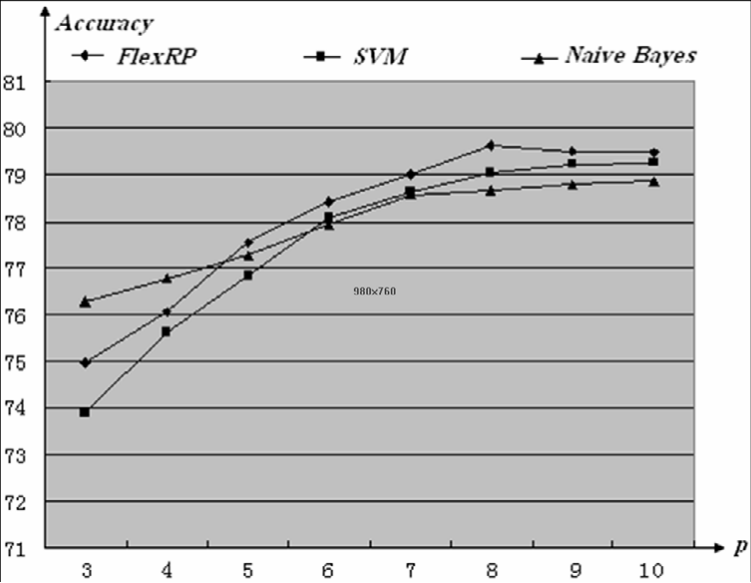
**The prediction accuracy in function of *p *for the *k*-spaced AA pairs where *k *≤ *p***. The number of features used to represent the sequence increases with the increasing value of *p*.

Additionally, we studied the impact of the varying values of the maximal spread, *k*, of the *k*-spaced AA pairs that are used to represent protein sequences on the prediction accuracy of the three optimized prediction methods. The accuracy in function of *p *for the *k*-spaced AA pairs where *k *≤ *p *and *p *= 3, 4, 5, 6, 7, 8, 9, 10 is shown in Figure 2. The results show that accuracy increases steadily for *p *between 3 and 8, and saturates above the latter value. The best accuracy corresponds to *p *= 8 and is achieved by the FlexRP, while on average, over the three methods, the accuracy for *p *= 8 equals 79.1%, and for *p *= 9 and 10 is higher and equals 79.2%. Therefore, the proposed sequence representation includes features for *p *= 9 (for *p *= 10 the accuracy is the same, but the number of features is larger).

### Comparison with similar prediction methods

The FlexRP was also compared with two recent methods that address similar predictions. Boden's group developed a method to predict regions that undergo conformational change via predicted continuum secondary structure [29]. On the other hand, the IUPred method performs prediction of intrinsically disordered/unstructured regions based on estimated energy content [30]. We note that although the above two methods perform similar prediction tasks, the definition of the flexible regions defined in this paper is different. Both of the above methods were tested on the same data as the FlexRP method and the results are summarized in Table 4. A direct comparison between accuracies may not be fair; however, low values of MCC for both IUPred and Boden's method in comparison with the MCC value for the FlexRP indicate that the proposed method is better suited for the prediction of flexible regions, as defined in this paper. The IUPred in general struggles with prediction of flexible regions, i.e., low sensitivity shows that it classifies a low number of the actual flexible residues as flexible, and low specificity shows that it classifies a relatively large number of the rigid residues as flexible, while doing relatively well in the case of prediction of the rigid regions. On the other hand, Boden's method is better balanced between the flexible and the rigid regions but it still overpredicts the flexible regions, i.e., it achieves low specificity for the flexible regions. The FlexRP method obtains relatively good predictions for both flexible and rigid regions.

**Figure 3:**
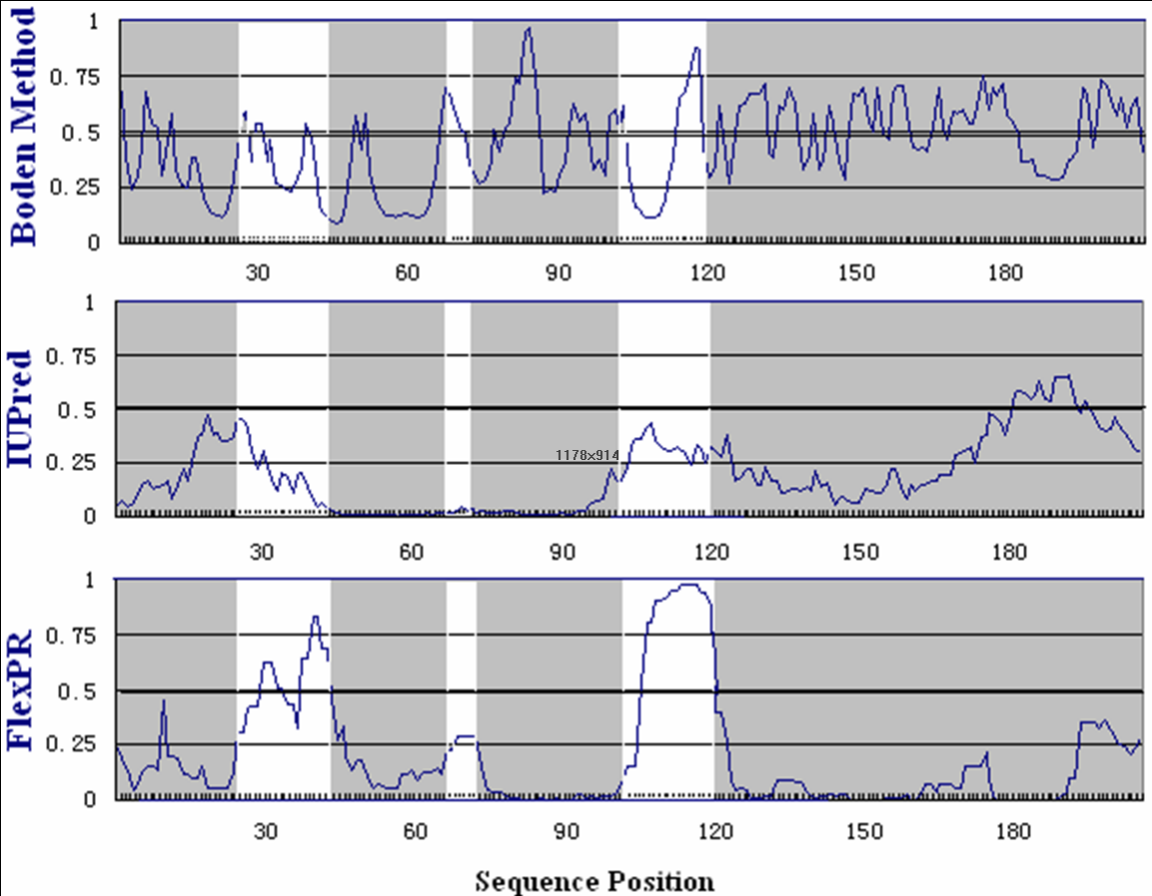
**The predictions obtained with the Boden's method [29], the IUPred method [30] and the FlexRP method on the 11E to 216A segment in chain A of 1EUL protein**. In the Boden's method residues with entropy greater than 0.49 are considered as regions undergoing conformational change; the IUPred method predicts all residues for which the probabilistic score is greater than 0.5 as belonging to the disordered regions. FlexRP classifies a residue as belonging to a flexible region if its corresponding probabilistic score is greater than 0.5. The actual flexible regions are identified using the white background.

We use an example to further demonstrate differences between the three prediction methods. The prediction was performed for a segment between 11E and 216A in chain A of 1EUL protein, see Figure 3. The continuum secondary structure is predicted by a cascaded probabilistic neural network (CPNN) [35], and the threshold to distinguish between the flexible and rigid residues is set to 0.49. The IUPred method uses a probabilistic score ranging between 0 (complete order) and 1 (total disorder), which is based on an energy value calculated using a pairwise energy profile along the sequence. This method uses a threshold that equals 0.5 to distinguish the disorder and ordered regions. Similar to the IUPred, the FlexRP method computes a probabilistic score that ranges between 0 (fully rigid) and 1 (fully flexible) and uses a threshold that equals 0.5.

In Figure 3, the actual (true) flexible regions are identified by the white background. Boden's method captures flexibility in all three flexible regions, but it also predicts over 50% of this sequence as flexible. This method performs prediction based on the entropy of the predicted secondary structure, and thus the quality of the predicted secondary structure determines the prediction of a flexible region. If CPNN is used with a sequence that shares low homology with the sequences that were used to train this neural network, then the resulting entropy may possibly have relatively large values, and as a result the corresponding residues will be classified as flexible (undergoing conformational change). Therefore, a large value of entropy may be related to the actual flexibility, or can be an artifact of a training set that does not include sufficiently homologous sequences. IUPred method generates scores that form local maxima around the first and the third flexible regions. However, the threshold is too high to identify them as disordered regions. We believe that this method could provide better prediction if a suitable optimization of the threshold value for a given sequence would be performed. At the same time, such optimization was not attempted in this method and may prove difficult to perform. Finally, the FlexRP method provides successful prediction of the first and the third flexible regions but it still misses the second, short flexible region that consists of 6 residues.

## Conclusion

Knowledge of flexibility/rigidity of protein sequence segments is of a pivotal role to improve the quality of the tertiary structure prediction methods and to attempt to fully solve the mystery of the protein folding process. At the same time, such information requires a very detailed knowledge of protein structure, and thus is available only for a small number of proteins. To this end, we propose a novel method, called FlexRP, for prediction of flexible/rigid regions based on protein sequence. The method is designed and tested using a set of segments for which flexibility/rigidity is defined based on a comprehensive exploration of tertiary structures from PDB [12]. It uses a novel protein sequence representation, which is based on 95 features computed as a frequency of selected k-spaced AA pairs, and a logistic regression classifier. Based on out-of-sample, 10-fold cross validation tests, the FlexRP is shown to predict the flexible/rigid regions with 80% accuracy, which may find practical applications. Finally, the proposed method is shown to be more accurate when compared with four other machine learning based approaches and two recently proposed methods that address similar prediction tasks.

## Methods

### Dataset

Our previous study that concerns conservation of the tertiary protein structures shows that less than 2% out of 8127 representative segments extracted from the entire Protein Data Bank (PDB) [36] have flexible tertiary structure [12]. The representative segments include the longest sequence segments that occur in multiple structures; as such they form a complete dataset to study the conservation. These 8127 segments were derived from release #103 of PDB that included a total of about 53000 protein chains. We first collected all sequence segments which were longer than 10 AAs and which occurred in at least two chains. After filtering out segments that were contained in longer segments, 8127 of them were kept, and we found that among them, 159 incorporated either a rotating or disarranging flexible region. Based on a visual inspection of the 159 segments, 66 and 93 of them were found to be *rotating *and *disarranging *segments, respectively. After removing short segments that include less than 50 AAs and segments that include flexible regions only at the head or the tail, we created a dataset of 66 sequences, which is used to develop and test the proposed prediction method, see Table 5. While this table lists only one representative protein that includes a given segment, a comprehensive database that includes information about the location of each segment in multiple chains, the sequence itself, and the annotation of flexible/rigid residues in this segment can be found at [37]. This dataset includes segments that are characterized by different structures which were obtained experimentally. Since the dataset is relatively small, the evaluation of the prediction is performed using 10-fold cross validation to avoid overfitting and to assure statistical validity of the computed quality indices.

### Definition of the flexible regions

Several different definitions of the flexible regions were proposed in the past:

1. all regions with NMR chemical shifts of a random-coil; regions that lack significantly ordered secondary structure (as determined by CD or FTIR); and/or regions that show hydrodynamic dimensions close to those typical of an unfolded polypeptide chain [38]

2. all regions with missing coordinates in X-ray structures [39,40]

3. stretches of 70 or more sequence-consecutive residues depleted of helices and strands [41]

4. regions with high B factors (normalized) from X-ray structures [42]

In this paper, a data-driven definition of flexible regions, which is based on a comprehensive exploration of the experimental protein structures, is proposed. A given sequence (region) is considered flexible if it has multiple different experimental structures (in different proteins), i.e. the corresponding structure is not conserved. Although two existing methods, i.e., FlexProt [43] and FatCat [44], can be used for identification of flexible regions for a pair of protein structures, a simpler and faster method that gives similar results was used. The selection of the applied method was motivated by the properties of the data that was used to define flexible regions, i.e., some segments in our dataset have dozens of structures and all combinations of pairs of structures had to be compared. Based on [12], the *flexible regions *are found using a sliding, six residues wide window. A protein sequence (segment) with *n *residues, which is denoted as *A*_1_*A*_2_...*A*_*i*-1_*A*_*i*_*A*_*i*+1_...*A*_*n*_, consists of following *n*-5 six-residue fragments

*A*_1_*A*_2_...*A*_5_*A*_6_, *A*_2_*A*_3_...*A*_6_*A*_7_,...,..., *A*_*n*-6 _*A*_*n*-5_...*A*_*n*-2_*A*_*n*-1_, *A*_*n*-5 _*A*_*n*-4_...*A*_*n*-1 _*A*_*n*_

The flexible regions were identified by comparing distance, which was computed using the Root Mean Square Distance for Unit Vectors (URMSD) measure [45], between structures of the six-residue fragments among the multiple structures that correspond to the same segments (see "Dataset" section). Let us assume that a given segment has *m *structures, which are stored in PDB. For convenience, the *m *structures of the corresponding *i*^th ^six-residue segment are denoted as *S*_*i*,1_, *S*_*i*,2_,..., *S*_*i*, *m*-1_, *S*_*i*, *m*_. Based on results in [12], if the URMSD between two structures is smaller or equal to 0.5, then they are assumed to be structurally similar; otherwise they are assumed to be different. Therefore, given that

max⁡1≤p<q≤mURMSD(Si,p,Si,q)>0.5
 MathType@MTEF@5@5@+=feaafiart1ev1aaatCvAUfKttLearuWrP9MDH5MBPbIqV92AaeXatLxBI9gBaebbnrfifHhDYfgasaacH8akY=wiFfYdH8Gipec8Eeeu0xXdbba9frFj0=OqFfea0dXdd9vqai=hGuQ8kuc9pgc9s8qqaq=dirpe0xb9q8qiLsFr0=vr0=vr0dc8meaabaqaciaacaGaaeqabaqabeGadaaakeaadaWfqaqaaiGbc2gaTjabcggaHjabcIha4bWcbaGaeGymaeJaeyizImQaemiCaaNaeyipaWJaemyCaeNaeyizImQaemyBa0gabeaakiabdwfavjabdkfasjabd2eanjabdofatjabdseaejabcIcaOiabdofatnaaBaaaleaacqWGPbqAcqGGSaalcqWGWbaCaeqaaOGaeiilaWIaem4uam1aaSbaaSqaaiabdMgaPjabcYcaSiabdghaXbqabaGccqGGPaqkcqGH+aGpcqaIWaamcqGGUaGlcqaI1aqnaaa@50E5@

the *i*^th ^six-residue fragments is defined as flexible; otherwise it is regarded as rigid. In other words, the regions characterized by maximal URMSD that are larger than 0.5 are indexed as flexible, while the remaining regions are indexed as rigid. The 66 segments that constitute our dataset include a total of 5716 residues, out of which 3929 were assumed as rigid and 1787 as flexible.

Following, we use an example, in which we aim to identify the flexible regions for 88G to 573R segment in chain A of 1UAA protein and chain B of 1UAA protein, to contrast results of the above method with the results of FlexPro and FatCat. Computation of flexible regions took 10 seconds for FlexProt, 30 seconds for Fatcat, and less than a second for the method that was used in this paper. The FlexProt identified a flexible region (hinge) between GLY374 and THR 375, the FatCat gave the same result, and the third method identified TYR369 to PHE 377 as the flexible region. While similar flexible regions were identified by all three methods, the method from [12] is an order of magnitude faster. The efficiency is especially crucial considering that most of the sequence segments had numerous structures and the computations had to be performed for all combinations of pairs of structures.

### FlexRP method

The proposed method performs its prediction as follows:

1. Each residue that constitutes the input sequence is represented by a feature vector. First, a 19-residues wide window, which is centered on the residue, is established. Next, frequencies of the 95 k-spaced AA pairs given in Table 2, which are inside the window, are computed.

2. The vector is inputted into a multinomial logistic regression model to predict if the residue should be classified as flexible or rigid.

The evaluation procedure applied in this paper assumes that the original dataset is divided into two disjoint sets: a training set that is used to develop the regression model and a test set that is used to test the quality of the proposed method (and other, considered methods). The logistic regression model is established through a Quasi-Newton optimization based on the training set [46]. Next, we provide details concerning the sequence representations and the performed experimental procedure.

### Feature-based sequence representation

Four representations, which include PSI-BLAST profile, composition vector, binary encoding, and the proposed collocation based features are applied to test and compare the quality of the proposed FlexRP method. A window that is centered on an AA for which the prediction is computed is used to compute the representation. In this paper, the window size is set to 19, i.e., the central AA and nine AAs on both of its sides. The size was selected based on a recent study that shows that such a window includes information required to predict and analyze folding of local structures and provides optimal results for secondary structure prediction [32].

The *composition vector *is a simple representation that is widely used in the prediction of various structural aspects [47-49]. Given that the 20 AAs, which are ordered alphabetically (*A*, *C*,..., *W*, *Y*), are represented as *AA*_1_, *AA*_2_,..., *AA*_19_, and *AA*_20_, and the number of occurrences of *AA*_i _in the local sequence window of size *k *(*k *= 19) is denoted as *n*_i_, the composition vector is defined as

(n1k,n2k,⋯⋯,n19k,n20k)
 MathType@MTEF@5@5@+=feaafiart1ev1aaatCvAUfKttLearuWrP9MDH5MBPbIqV92AaeXatLxBI9gBaebbnrfifHhDYfgasaacH8akY=wiFfYdH8Gipec8Eeeu0xXdbba9frFj0=OqFfea0dXdd9vqai=hGuQ8kuc9pgc9s8qqaq=dirpe0xb9q8qiLsFr0=vr0=vr0dc8meaabaqaciaacaGaaeqabaqabeGadaaakeaacqGGOaakdaWcaaqaaiabd6gaUnaaBaaaleaacqaIXaqmaeqaaaGcbaGaem4AaSgaaiabcYcaSmaalaaabaGaemOBa42aaSbaaSqaaiabikdaYaqabaaakeaacqWGRbWAaaGaeiilaWIaeS47IWKaeS47IWKaeiilaWYaaSaaaeaacqWGUbGBdaWgaaWcbaGaeGymaeJaeGyoaKdabeaaaOqaaiabdUgaRbaacqGGSaaldaWcaaqaaiabd6gaUnaaBaaaleaacqaIYaGmcqaIWaamaeqaaaGcbaGaem4AaSgaaiabcMcaPaaa@4794@

Another popular protein sequence representation is based on *binary encoding *[50,51]. In this case, a vector of 20 values is used to encode each AA. For *AA*_i_, the *i*^th ^position of the vector is set to 1, and the remaining 19 values are set to 0. Each of the AAs in the local sequence window of size *k *= 19 is represented by such a vector, and the combined vector of 19*20 = 380 features is used to predict flexibility/rigidity of the central AA.

*PSI-BLAST profile *[52] is one of the most commonly used representations in a variety of prediction tasks related to proteins [35,51,53]. Using a PSI-BLAST method, a target protein sequence is first (multiply-) aligned with orthologous sequences. *X*_*i *_is set to the log-odds score vector (over the 20 possible AAs) derived from the multiple alignment column corresponding to the *i*^th ^position in the window. This method treats each *X*_*i *_as a 21-dimensional vector of real values; the extra dimension is used to indicate whether *X*_*i *_is off the end of the actual protein sequence (0 for within sequence, 0.5 for outside). The log-odds alignment scores are obtained by running PSI-BLAST against Genbank's standard non-redundant protein sequence database for three iterations. In this paper, PSI-BLAST profiles were run with default parameters and a window size of 15 as suggested in [35] and [53].

A new representation, which is based on frequency of *k-spaced *AA pairs in the local sequence window, was developed for the proposed prediction method. Our motivation was that the flexibility of each AA is different, i.e. AAs with smaller side chain (e.g. Glycine) may be structurally more flexible since they are less affected by the arrangement of the side chains of adjacent AAs. Furthermore, if several AAs that are characterized by potentially higher flexibility would cluster together, then the corresponding entire region (window) would be more likely to be flexible. Based on this argument, for a given central AA, a sliding sequence window of size *k *= 19 was used to count all adjacent pairs of AAs (dipeptides) in that window. Since there are 400 possible AA pairs (*AA*, *AC*, *AD*,..., *YY*), a feature vector of that size is used to represent occurrence of these pairs in the window. For instance, if an AG pair occurs four times in this window, the corresponding value in the vector is set to 4, while if a KN pair would not occur in the window, the corresponding value would be set to 0. Since short-range interactions between AAs, rather than only interactions between immediately adjacent AAs, have impact on folding [32], the proposed representation also considers k-spaced pairs of AAs, i.e. pairs that are separated by *p *other AAs. K-spaced pairs for *p *= 0, 1,..., 9 are considered, where for *p *= 0 the pairs reduce to dipeptides. For each value of *p*, there are 400 corresponding features. Table 6 compares the four representations with respect to their corresponding number of features.

### Feature selection

The binary encoding and the collocation based representations include relatively large number of features. Therefore, two selection methods, i.e., correlation and entropy based, were used to reduce the dimensionality and potentially improve the prediction accuracy by selecting a subset of the features.

The *correlation-based feature selection *is based on Pearson correlation coefficient *r *computed for a pair of variables (*X*, *Y*) [54] as

r=∑i(xi−xi_)(yi−yi_)∑i(xi−xi_)2∑i(yi−yi_)2
 MathType@MTEF@5@5@+=feaafiart1ev1aaatCvAUfKttLearuWrP9MDH5MBPbIqV92AaeXatLxBI9gBaebbnrfifHhDYfgasaacH8akY=wiFfYdH8Gipec8Eeeu0xXdbba9frFj0=OqFfea0dXdd9vqai=hGuQ8kuc9pgc9s8qqaq=dirpe0xb9q8qiLsFr0=vr0=vr0dc8meaabaqaciaacaGaaeqabaqabeGadaaakeaacqWGYbGCcqGH9aqpdaWcaaqaamaaqafabaGaeiikaGIaemiEaG3aaSbaaSqaaiabdMgaPbqabaGccqGHsisldaWfGaqaaiabdIha4naaBaaaleaacqWGPbqAaeqaaaqabeaacqGGFbWxaaGccqGGPaqkcqGGOaakcqWG5bqEdaWgaaWcbaGaemyAaKgabeaakiabgkHiTmaaxacabaGaemyEaK3aaSbaaSqaaiabdMgaPbqabaaabeqaaiabc+faFbaakiabcMcaPaWcbaGaemyAaKgabeqdcqGHris5aaGcbaWaaOaaaeaadaaeqbqaaiabcIcaOiabdIha4naaBaaaleaacqWGPbqAaeqaaOGaeyOeI0YaaCbiaeaacqWG4baEdaWgaaWcbaGaemyAaKgabeaaaeqabaGaei4xa8faaOGaeiykaKYaaWbaaSqabeaacqaIYaGmaaaabaGaemyAaKgabeqdcqGHris5aaWcbeaakmaakaaabaWaaabuaeaacqGGOaakcqWG5bqEdaWgaaWcbaGaemyAaKgabeaakiabgkHiTmaaxacabaGaemyEaK3aaSbaaSqaaiabdMgaPbqabaaabeqaaiabc+faFbaakiabcMcaPmaaCaaaleqabaGaeGOmaidaaaqaaiabdMgaPbqab0GaeyyeIuoaaSqabaaaaaaa@64FF@

where xi¯
 MathType@MTEF@5@5@+=feaafiart1ev1aaatCvAUfKttLearuWrP9MDH5MBPbIqV92AaeXatLxBI9gBaebbnrfifHhDYfgasaacH8akY=wiFfYdH8Gipec8Eeeu0xXdbba9frFj0=OqFfea0dXdd9vqai=hGuQ8kuc9pgc9s8qqaq=dirpe0xb9q8qiLsFr0=vr0=vr0dc8meaabaqaciaacaGaaeqabaqabeGadaaakeaadaqdaaqaaiabdIha4naaBaaaleaacqWGPbqAaeqaaaaaaaa@2FBD@ is the mean of *X*, and yi¯
 MathType@MTEF@5@5@+=feaafiart1ev1aaatCvAUfKttLearuWrP9MDH5MBPbIqV92AaeXatLxBI9gBaebbnrfifHhDYfgasaacH8akY=wiFfYdH8Gipec8Eeeu0xXdbba9frFj0=OqFfea0dXdd9vqai=hGuQ8kuc9pgc9s8qqaq=dirpe0xb9q8qiLsFr0=vr0=vr0dc8meaabaqaciaacaGaaeqabaqabeGadaaakeaadaqdaaqaaiabdMha5naaBaaaleaacqWGPbqAaeqaaaaaaaa@2FBF@ the mean of *Y*. The value of *r *is bounded within the [-1, 1] interval. Higher absolute value of *r *corresponds to higher correlation between *X *and *Y*. The method computes the correlation coefficient between each feature (variable) and the known predicted variable, i.e. flexibility/rigidity values (based on the training data) and selects a subset of features that have the highest absolute *r *value.

The *entropy-based feature selection *is based on information theory, which defines entropy of a variable X as

H(X)=−∑iP(xi)log⁡2(P(xi))
 MathType@MTEF@5@5@+=feaafiart1ev1aaatCvAUfKttLearuWrP9MDH5MBPbIqV92AaeXatLxBI9gBaebbnrfifHhDYfgasaacH8akY=wiFfYdH8Gipec8Eeeu0xXdbba9frFj0=OqFfea0dXdd9vqai=hGuQ8kuc9pgc9s8qqaq=dirpe0xb9q8qiLsFr0=vr0=vr0dc8meaabaqaciaacaGaaeqabaqabeGadaaakeaacqWGibascqGGOaakcqWGybawcqGGPaqkcqGH9aqpcqGHsisldaaeqbqaaiabdcfaqjabcIcaOiabdIha4naaBaaaleaacqWGPbqAaeqaaOGaeiykaKIagiiBaWMaei4Ba8Maei4zaC2aaSbaaSqaaiabikdaYaqabaGccqGGOaakcqWGqbaucqGGOaakcqWG4baEdaWgaaWcbaGaemyAaKgabeaakiabcMcaPiabcMcaPaWcbaGaemyAaKgabeqdcqGHris5aaaa@48E3@

where {*x*_*i*_} is a set of values of *X *and *P*(*x*_*i*_) is the prior probability of *x*_*i*_.

The conditional entropy of *X*, given another variable *Y *is defined as

H(X|Y)=−∑jP(yj)∑iP(xi|yj)log⁡2(P(xi|yj))
 MathType@MTEF@5@5@+=feaafiart1ev1aaatCvAUfKttLearuWrP9MDH5MBPbIqV92AaeXatLxBI9gBaebbnrfifHhDYfgasaacH8akY=wiFfYdH8Gipec8Eeeu0xXdbba9frFj0=OqFfea0dXdd9vqai=hGuQ8kuc9pgc9s8qqaq=dirpe0xb9q8qiLsFr0=vr0=vr0dc8meaabaqaciaacaGaaeqabaqabeGadaaakeaacqWGibascqGGOaakcqWGybawcqGG8baFcqWGzbqwcqGGPaqkcqGH9aqpcqGHsisldaaeqbqaaiabdcfaqjabcIcaOiabdMha5naaBaaaleaacqWGQbGAaeqaaOGaeiykaKYaaabuaeaacqWGqbauaSqaaiabdMgaPbqab0GaeyyeIuoakiabcIcaOiabdIha4naaBaaaleaacqWGPbqAaeqaaOGaeiiFaWNaemyEaK3aaSbaaSqaaiabdQgaQbqabaGccqGGPaqkcyGGSbaBcqGGVbWBcqGGNbWzdaWgaaWcbaGaeGOmaidabeaakiabcIcaOiabdcfaqjabcIcaOiabdIha4naaBaaaleaacqWGPbqAaeqaaOGaeiiFaWNaemyEaK3aaSbaaSqaaiabdQgaQbqabaGccqGGPaqkcqGGPaqkaSqaaiabdQgaQbqab0GaeyyeIuoaaaa@5E2D@

where *P*(*x*_*i*_| *y*_*j*_) is the posterior probability of *X *given the value *y*_*i *_of *Y*.

The amount by which the entropy of *X *decreases reflects additional information about *X *provided by *Y *and is called information gain [54]

*IG*(*X *| *Y*) = *H*(*X*) - *H*(*X *| *Y*)

According to this measure, Y is regarded as more highly correlated with *X *than *Z *if *IG*(*X*|*Y*) > *IG*(*Z*|*Y*). Similar to the correlation-based selection, this method computes the information gain value between each feature (variable) and the known predicted variable, i.e. flexibility/rigidity values (based on the training data) and selects a subset of features that have the highest value of *IG*.

### Logistic regression

Logistic regression is a method suitable to model a relationship between a binary response variable and one or more predictor variables, which may be either discrete or continuous. As such, this model perfectly fits the data used in this paper, i.e., the response variable is a binary flexible/rigid classification of a residue, and the predictor variables are the frequency of the selected k-spaced AA pairs in the local sequence window. We applied a statistical regression model for Bernoulli-distributed dependent variables, which is implemented as a generalized linear model that utilizes the logit as its link function. The model takes the following form

log⁡it(pi)=ln⁡(pi1−pi)=α+β1x1,i+...+βkxk,i
 MathType@MTEF@5@5@+=feaafiart1ev1aaatCvAUfKttLearuWrP9MDH5MBPbIqV92AaeXatLxBI9gBaebbnrfifHhDYfgasaacH8akY=wiFfYdH8Gipec8Eeeu0xXdbba9frFj0=OqFfea0dXdd9vqai=hGuQ8kuc9pgc9s8qqaq=dirpe0xb9q8qiLsFr0=vr0=vr0dc8meaabaqaciaacaGaaeqabaqabeGadaaakeaacyGGSbaBcqGGVbWBcqGGNbWzcqWGPbqAcqWG0baDcqGGOaakcqWGWbaCdaWgaaWcbaGaemyAaKgabeaakiabcMcaPiabg2da9iGbcYgaSjabc6gaUjabcIcaOmaalaaabaGaemiCaa3aaSbaaSqaaiabdMgaPbqabaaakeaacqaIXaqmcqGHsislcqWGWbaCdaWgaaWcbaGaemyAaKgabeaaaaGccqGGPaqkcqGH9aqpiiGacqWFXoqycqGHRaWkcqWFYoGydaWgaaWcbaGaeGymaedabeaakiabdIha4naaBaaaleaacqaIXaqmcqGGSaalcqWGPbqAaeqaaOGaey4kaSIaeiOla4IaeiOla4IaeiOla4Iaey4kaSIae8NSdi2aaSbaaSqaaiabdUgaRbqabaGccqWG4baEdaWgaaWcbaGaem4AaSMaeiilaWIaemyAaKgabeaaaaa@5DAB@

where *i *= 1,..., *n*, *n *is the number of instances, and *P*_*i *_= *P*(*Y*_*i *_= 1).

The logarithm of the odds (probability divided by 1 – probability) of the outcome is modeled as a linear function of the predictor variables, *X*_*i*_. This can be written equivalently as

pi=P(Yi=1|X)=eα+β1x1,i+...+βkxk,i1+eα+β1x1,i+...+βkxk,i
 MathType@MTEF@5@5@+=feaafiart1ev1aaatCvAUfKttLearuWrP9MDH5MBPbIqV92AaeXatLxBI9gBaebbnrfifHhDYfgasaacH8akY=wiFfYdH8Gipec8Eeeu0xXdbba9frFj0=OqFfea0dXdd9vqai=hGuQ8kuc9pgc9s8qqaq=dirpe0xb9q8qiLsFr0=vr0=vr0dc8meaabaqaciaacaGaaeqabaqabeGadaaakeaacqWGWbaCdaWgaaWcbaGaemyAaKgabeaakiabg2da9iabdcfaqjabcIcaOiabdMfaznaaBaaaleaacqWGPbqAaeqaaOGaeyypa0JaeGymaeJaeiiFaWNaemiwaGLaeiykaKIaeyypa0ZaaSaaaeaacqWGLbqzdaahaaWcbeqaaGGaciab=f7aHjabgUcaRiab=j7aInaaBaaameaacqaIXaqmaeqaaSGaemiEaG3aaSbaaWqaaiabigdaXiabcYcaSiabdMgaPbqabaWccqGHRaWkcqGGUaGlcqGGUaGlcqGGUaGlcqGHRaWkcqWFYoGydaWgaaadbaGaem4AaSgabeaaliabdIha4naaBaaameaacqWGRbWAcqGGSaalcqWGPbqAaeqaaaaaaOqaaiabigdaXiabgUcaRiabdwgaLnaaCaaaleqabaGae8xSdeMaey4kaSIae8NSdi2aaSbaaWqaaiabigdaXaqabaWccqWG4baEdaWgaaadbaGaeGymaeJaeiilaWIaemyAaKgabeaaliabgUcaRiabc6caUiabc6caUiabc6caUiabgUcaRiab=j7aInaaBaaameaacqWGRbWAaeqaaSGaemiEaG3aaSbaaWqaaiabdUgaRjabcYcaSiabdMgaPbqabaaaaaaaaaa@6EFE@

In contrast to the linear regression, in which parameters *α*, *β*_1_,..., *β*_*k *_are calculated using minimal squared error, parameters in the logistic regression are usually estimated by maximum likelihood. More specifically, (*α*, *β*_1_,..., *β*_*k*_) is a set of values that maximizes the following likelihood function

L(α,β1,β2,...,βk)=∏i=1npiyi(1−pi)1−yi
 MathType@MTEF@5@5@+=feaafiart1ev1aaatCvAUfKttLearuWrP9MDH5MBPbIqV92AaeXatLxBI9gBaebbnrfifHhDYfgasaacH8akY=wiFfYdH8Gipec8Eeeu0xXdbba9frFj0=OqFfea0dXdd9vqai=hGuQ8kuc9pgc9s8qqaq=dirpe0xb9q8qiLsFr0=vr0=vr0dc8meaabaqaciaacaGaaeqabaqabeGadaaakeaacqWGmbatcqGGOaakiiGacqWFXoqycqGGSaalcqWFYoGydaWgaaWcbaGaeGymaedabeaakiabcYcaSiab=j7aInaaBaaaleaacqaIYaGmaeqaaOGaeiilaWIaeiOla4IaeiOla4IaeiOla4IaeiilaWIae8NSdi2aaSbaaSqaaiabdUgaRbqabaGccqGGPaqkcqGH9aqpdaqeWbqaaiabdchaWnaaDaaaleaacqWGPbqAaeaacqWG5bqEdaWgaaadbaGaemyAaKgabeaaaaGccqGGOaakcqaIXaqmcqGHsislcqWGWbaCdaWgaaWcbaGaemyAaKgabeaakiabcMcaPmaaCaaaleqabaGaeGymaeJaeyOeI0IaemyEaK3aaSbaaWqaaiabdMgaPbqabaaaaaWcbaGaemyAaKMaeyypa0JaeGymaedabaGaemOBa4ganiabg+Givdaaaa@5989@

### Experimental setup

The classification systems used to develop and compare the proposed systems were implemented in Weka, which is a comprehensive open-source library of machine learning methods [55]. The proposed FlexRP method applies multinomial logistic regression [46]. Our method was compared with a state-of-the-art Support Vector Machine classifier [34], popular and simple Naïve Bayes classifier [33], instance learning based IB1 classifier [56] and C4.5 decision tree classifier [57]. The experimental evaluation was performed using 10-fold cross validation to avoid overfitting and assure statistical validity of the results. To avoid overlap (with respect to sequences) between training and test sets, the entire set of 66 sequences is divided into 10 folds, i.e. 6 folds that include 7 sequences and 4 folds with 6 sequences. In 10-fold cross validation, 9 folds together are used as a training data to generate the prediction model and the remaining, set aside, fold is used for testing. The test is repeated 10 times, each time using a different fold as the test set.

The reported results include the following quality indices:

Sensitivity=TPTP+FNSpecificity=TNTN+FP
 MathType@MTEF@5@5@+=feaafiart1ev1aaatCvAUfKttLearuWrP9MDH5MBPbIqV92AaeXatLxBI9gBaebbnrfifHhDYfgasaacH8akY=wiFfYdH8Gipec8Eeeu0xXdbba9frFj0=OqFfea0dXdd9vqai=hGuQ8kuc9pgc9s8qqaq=dirpe0xb9q8qiLsFr0=vr0=vr0dc8meaabaqaciaacaGaaeqabaqabeGadaaakeaafaqabeqacaaabaGaem4uamLaemyzauMaemOBa4Maem4CamNaemyAaKMaemiDaqNaemyAaKMaemODayNaemyAaKMaemiDaqNaemyEaKNaeyypa0ZaaSaaaeaacqWGubavcqWGqbauaeaacqWGubavcqWGqbaucqGHRaWkcqWGgbGrcqWGobGtaaaabaGaem4uamLaemiCaaNaemyzauMaem4yamMaemyAaKMaemOzayMaemyAaKMaem4yamMaemyAaKMaemiDaqNaemyEaKNaeyypa0ZaaSaaaeaacqWGubavcqWGobGtaeaacqWGubavcqWGobGtcqGHRaWkcqWGgbGrcqWGqbauaaaaaaaa@5C95@

Accuracy=TP+TNTP+TN+FP+FNMCC=TP×TN−FP×FN(TP+FP)(TP+FN)(TN+FP)(TN+FN)
 MathType@MTEF@5@5@+=feaafiart1ev1aaatCvAUfKttLearuWrP9MDH5MBPbIqV92AaeXatLxBI9gBaebbnrfifHhDYfgasaacH8akY=wiFfYdH8Gipec8Eeeu0xXdbba9frFj0=OqFfea0dXdd9vqai=hGuQ8kuc9pgc9s8qqaq=dirpe0xb9q8qiLsFr0=vr0=vr0dc8meaabaqaciaacaGaaeqabaqabeGadaaakeaafaqabeqacaaabaGaemyqaeKaem4yamMaem4yamMaemyDauNaemOCaiNaemyyaeMaem4yamMaemyEaKNaeyypa0ZaaSaaaeaacqWGubavcqWGqbaucqGHRaWkcqWGubavcqWGobGtaeaacqWGubavcqWGqbaucqGHRaWkcqWGubavcqWGobGtcqGHRaWkcqWGgbGrcqWGqbaucqGHRaWkcqWGgbGrcqWGobGtaaaabaGaemyta0Kaem4qamKaem4qamKaeyypa0ZaaSaaaeaacqWGubavcqWGqbaucqGHxdaTcqWGubavcqWGobGtcqGHsislcqWGgbGrcqWGqbaucqGHxdaTcqWGgbGrcqWGobGtaeaadaGcaaqaaiabcIcaOiabdsfaujabdcfaqjabgUcaRiabdAeagjabdcfaqjabcMcaPiabcIcaOiabdsfaujabdcfaqjabgUcaRiabdAeagjabd6eaojabcMcaPiabcIcaOiabdsfaujabd6eaojabgUcaRiabdAeagjabdcfaqjabcMcaPiabcIcaOiabdsfaujabd6eaojabgUcaRiabdAeagjabd6eaojabcMcaPaWcbeaaaaaaaaaa@7922@

where TP, TN, FP and FN denote true positive, true negative, false positive and false negative, respectively.

## Authors' contributions

KC and LK developed the prediction method and performed the experimental evaluation. KC and JR contributed to the data collection and definition of flexible regions. All authors contributed to writing the manuscript, and read and approved the final version.

## Figures and Tables

**Table 1:** Prediction accuracy for different protein sequence representations based on 10-fold cross validation tests.

Feature representation	Classifier^1 ^Feature selection^2^	FlexRP (Logistic Regression)	SVM	C4.5	IB1	Naïve Bayes
Composition vector	N/A	67.37%	68.74%	57.70%	57.33%	65.20%
PSI-BLAST profile	N/A	66.38%	67.35%	62.47%	61.62%	66.24%
Binary encoding	No selection	66.38%	66.06%	58.82%	59.92%	61.84%
Binary encoding	Linear coefficient	69.58%	68.74%	62.82%	57.05%	69.10%
Binary encoding	Entropy based	69.19%	68.74%	63.24%	58.21%	69.00%
K-spaced AA pairs	Linear coefficient	74.37%	74.60%	66.04%	68.74%	72.97%
K-spaced AA pairs	Entropy based	**79.51**%^3^	78.46%	66.25%	66.93%	76.01%

^1^The tested classifiers include the proposed FlexRP method, Support Vector Machine (SVM), decision tree (C4.5), instance-based learner (IB1), and Naïve Bayes.

^2 ^The sequence representations based on binary codes and frequencies of the k-spaced amino acid pairs were processed using two feature selection methods.

^3 ^The best result is shown in bold.

**Table 2:** Features selected by the entropy based method.

*k*-spaced AA pairs^1^									
*k *= 0	*k *= 1	*k *= 2	*k *= 3	*k *= 4	*k *= 5	*k *= 6	*k *= 7	*k *= 8	*k *= 9

DF	AK	DI	AD	AI	DC	DI	ED	DP	AC
EF	FH	ED	AI	AV	HD	FF	GL	EN	EL
EL	KI	EK	AV	AY	IE	FG	PG	GG	KF
KE	KY	FK	GG	DG	NQ	HP	PS	KC	KG
LI	LL	GG	KQ	DS	PG	IL	TI	RI	
LL	LQ	GR	LI	EK	QQ	QP	VI	TV	
PA	PM	GS	LS	ER	RV	TL	VN	VR	
QT	VH	KL	PW	HQ	VL	VR			
VI	VL	KS	SG	LL	VV	YC			
VP		LL	YH	LV	YL				
			PS		MV				
			QQ		PD				
			VI		SQ				
			VK		TK				
					VL				

^1^*k*-spaced AA pairs represent frequency of the AA pairs that are separated by *k *other residues in the sequence; for k = 0 the pairs are equivalent to dipeptides.

**Table 3:** Prediction accuracy after optimization.

		*rigid *regions		*flexible *regions						
							
Method	Accuracy^1^	sensitivity	specificity	sensitivity	specificity	MCC	TP	FP	FN	TN
FlexRP	79.51%	88.52%	82.85%	59.71%	70.24%	0.51	3478	720	451	1067
SVM	79.22%	88.93%	82.27%	57.86%	70.39%	0.50	3494	753	435	1034
Naïve Bayes	78.41%	80.15%	87.40%	74.59%	63.09%	0.53	3149	454	780	1333

^1 ^The results were based on the best performing representation that includes 95 features selected using the entropy based selection method.

**Table 4:** Comparison of performances between FlexRP, IUPred, and Boden's methods.

		*rigid *regions		*flexible *regions						
							
Method	Accuracy	sensitivity	specificity	sensitivity	specificity	MCC	TP	FP	FN	TN
FlexRP	79.51%	88.52%	82.85%	59.71%	70.24%	0.51	3478	720	451	1067
IUPred	65.64%	88.88%	69.58%	14.55%	37.30%	0.05	3492	1527	437	260
Boden's method	56.21%	56.71%	73.53%	55.12%	36.67%	0.11	2228	802	1701	985

**Table 5:** List of 66 segments with multiple experimental structures.

Protein ID^1^	Start AA	End AA	Protein ID^1^	Start AA	End AA	Protein ID^1^	Start AA	End AA
1eulA	11E	216A	1c0mA	199K	268D	1ic8A	208P	276A
121p	25Q	166H	1cdb	24F	105R	1ihgA	245K	298E
1a0h	482V	575D	1cejA	30C	95S	1iku	104W	189E
1a7lA	4E	198L	1cfpA	2E	80I	1ilf	7D	140Q
1a7xA	31E	106L	1cpq	7L	128E	1irf	27L	112L
1a90	25L	116Q	1cto	46R	108M	1jmvA	68Q	139R
1ael	41T	111N	1dem	4R	59R	1k0tA	11I	80Y
1akk	34G	103N	1dhx	339A	430G	1k9aA	117T	316A
1al01	42V	124T	1dmzA	613I	706G	1kmuR	299E	382Y
1aonA	218P	371K	1do0	42E	165E	1kvnA	23I	89R
1ap9	104D	155G	1ei7A	60V	148S	1l6kA	8E	61V
1avfJ	76G	155L	1ej6B	723A	928V	1mfn	3D	184T
1az0A	191S	244R	1f2hA	59L	164C	1mkmA	10I	215S
1b4m	42I	134K	1ffxA	148G	263P	1o0vA	265Q	470M
1b75A	41E	94A	1fm6A	266T	430L	1pbwA	238L	297E
1b7eA	133E	239I	1g3gA	44P	152K	1qpmA	28M	81T
1b8tA	12V	191S	1gm0	15A	122I	1sw6A	346Y	429S
1ba9	72G	123A	1go4G	498F	578M	1uaaA	88G	537R
1blr	41V	96E	1hqmD	1039L	1116T	1wtuA	14T	99K
1boc	6L	75Q	1hryA	20R	75R	2btfA	4D	71I
1bqmA	276V	400L	1hstA	26P	79G	2ezm	1L	100Y
1bsh	19L	138M	1i84S	883E	942E	5gcn	36M	165G

^1 ^For each segment, one PDB ID together with the start and the end of the segment are listed.

**Table 6:** Sizes of feature sets for the considered sequence representations.

				k-spaced AA pairs				
				
Feature representation	Composition Vector	PSI-BLAST profile	Binary Encoding	adjacent pairs (dipeptides)	1-spaced pairs	......	*p*-spaced pairs	Total
Number of features	20	315	380	400	400	......	400	400(*p*+1)
